# The Actions of Centrally Administered Nesfatin-1 on Emesis, Feeding, and Locomotor Activity in *Suncus murinus* (House Musk Shrew)

**DOI:** 10.3389/fphar.2022.858522

**Published:** 2022-04-06

**Authors:** Zengbing Lu, Dexuan Cui, Julia Yuen Hang Liu, Bin Jiang, Man Piu Ngan, Ichiro Sakata, Shota Takemi, Takafumi Sakai, Ge Lin, Sze Wa Chan, John A. Rudd

**Affiliations:** ^1^ School of Biomedical Sciences, The Chinese University of Hong Kong, Hong Kong, Hong Kong SAR, China; ^2^ School of Health Sciences, Caritas Institute of Higher Education, Hong Kong, Hong Kong SAR, China; ^3^ Graduate School of Science and Engineering, Saitama University, Saitama, Japan; ^4^ The Laboratory Animal Services Centre, The Chinese University of Hong Kong, Hong Kong, Hong Kong SAR, China

**Keywords:** emesis, food intake, nesfatin-1, NUCB2, *Suncus murinus*

## Abstract

Nesfatin-1 is an anorectic peptide expressed in both peripheral tissues and brain areas involved in the regulation of feeding, emotion and emesis. The aim of the present study is to characterize the distribution of NUCB2/nesfatin-1 in *Suncus murinus* and to investigate the actions of nesfatin-1 to affect gastrointestinal contractility, emesis, food and water intake, and locomotor activity. The deduced amino acid sequence of *S. murinus* nesfatin-1 using *in silico* cloning showed high homology with humans and rodents. NUCB2 mRNA was detected throughout the entire brain and in the gastrointestinal tract, including the stomach and gut. Western blot analysis and immunohistochemistry confirmed the expression of nesfatin-1 protein in these regions. The NUCB2 mRNA levels in the hypothalamus, hippocampus and brainstem were significantly decreased, whereas that in the striatum were increased after 24 h starvation compared to *ad libitum*-fed animals (*p* < 0.05). In *in vitro* studies, nesfatin-1 (0.3–1,000 pM) failed to contract or relax the isolated gastric antrum and intestinal segments. In conscious, freely moving animals, intracerebroventricular administration of nesfatin-1 (1–50 pmol) induced emesis (*p* < 0.05) and suppressed 6-h cumulative food intake (*p* < 0.05), without affecting the latency to feeding. Nesfatin-1 (25 pmol, i.c.v.) decreased 24-h cumulative food and water intake by 28.3 and 35.4%, respectively (*p* < 0.01). No significant differences in locomotor activity were observed. In conclusion, NUCB2/nesfatin-1 might be a potent regulator of feeding and emesis in *S. murinus*. Further studies are required to elucidate the mechanism of actions of this peptide as a mediator linking the brainstem NUCB2/nesfatin-1 to forebrain system.

## Introduction

The gut–brain axis plays a critical role in the homeostatic regulation of energy balance and appetite (Hussain and Bloom, 2013). Numerous peptides that act as neurotransmitters/neuromodulators and act at both peripheral and central receptors modulate appetite and gastric emptying may possibly be involved in mechanism regulating the sensation of nausea and emesis (Noetzel et al., 2009; Chan et al., 2013; Rudd et al., 2018; Lu et al., 2020). The hypothalamus integrates signals to regulate feeding and glucose homoeostasis (Oh et al., 2006; Yang et al., 2012), whereas the brainstem is involved in the autonomic regulation of visceral-endocrine functions (Goebel et al., 2009) and the mechanisms of nausea and emesis (Lu et al., 2017).

Nesfatin-1 is an 82-amino acid anorectic peptide derived from nucleobindin2 (NUCB2), a 396-amino acid protein that is highly conserved in humans, rats and mice (Oh et al., 2006; Mohan and Unniappan, 2013). While post-translational processing of NUCB2 results in nesfatin-1 (aa 1-82), nesfatin-2 (aa 85-163) and nesfatin-3 (aa 166-369), only nesfatin-1 has biological activity (Oh et al., 2006). NUCB2/nesfatin-1 immunoreactivity are expressed in peripheral tissues i.e., the stomach, pancreas and adipose tissues (Zhang et al., 2010) and the brain, including the cortical areas, the limbic system, thalamus, hypothalamus as well as medullary nuclei such as the nucleus tractus solitarius (NTS) and dorsal motor nucleus of the vagus (DMNV), (Oh et al., 2006; Foo et al., 2008; Goebel-Stengel and Wang, 2013), and these brain areas have been implicated in feeding, emotion and emesis (Oh et al., 2006; Maejima et al., 2009; Goebel-Stengel et al., 2011).

Intracerebroventricular administration of nesfatin-1 potently reduces dark-phase food intake (Oh et al., 2006; Maejima et al., 2009). Conversely, intracerebral third ventricular administration of a NUCB2 antisense oligonucleotide increases body weight gain (Oh et al., 2006). Furthermore, there is growing evidence that NUCB2/nesfatin-1 is implicated in the long-term modulation of energy balance, making it a promising target for drug treatment of obese patients (Stengel et al., 2011). However, despite the increasing knowledge on the roles of nesfatin-1, its corresponding receptor has not yet been identified. Up to now, the potential involvement of nesfatin-1 in emesis control is less well understood because common laboratory animals (e.g., rat and mouse) are incapable of emesis and therefore the role of nesfatin-1 as a transmitter linking the forebrain and brainstem being involved in feeding and the control of nausea and emesis has been overlooked.

In the present study, therefore, we deduced the amino acid sequence of *Suncus murinus* NUCB2/nesfatin-1 using *in silico* cloning. We identified the expression of NUCB2 mRNA using reverse transcription PCR and investigated its levels of expression in the fed and 24 h fasted states using real-time PCR. The distribution and protein expression of nesfatin-1 peptide were determined using immunohistochemistry and western blotting, respectively. We then investigated the effect of nesfatin-1 on isolated gastric antrum and gut regions. Finally, we investigated the action of nesfatin-1 following intracerebroventricular administration to affect locomotor activity, feeding and emesis in conscious, freely moving animals.

## Materials and Methods

### Animals

Male *S. murinus* (45–70 g) was purchased from the Laboratory Animal Services Centre of the Chinese University of Hong Kong. Animals were housed in a temperature-controlled room at 24 ± 1 °C with humidity at 50 ± 5% and lights on 0600–1800 h. Water and dry pelleted cat chow (Feline Diet 5003, PMI^®^ Feeds, St. Louis, United States) were given *ad libitum*. The Feline Diet 5003 has been used in our group and others in research investigating feeding and emesis (Rudd et al., 1999; Chan et al., 2013; Borner et al., 2020). All the animal experiments were performed under license from the Government of Hong Kong SAR and under the approval of the Animal Experimentation Ethics Committee of the Chinese University of Hong Kong.

### *In Silico* Analysis

Nesfatin-1 amino acid sequence of *S. murinus* was deduced from the *S. murinus* genome browser. Amino acid sequence from various species, including human, rats and mice were obtained from GenBank.

### Reverse Transcription PCR (RT-PCR)

Three *S. murinus* were euthanized by CO_2_ anesthesia followed by cervical dislocation. Stomach, duodenum, ileum, colon, and brain tissues including the olfactory bulb, cortex, striatum, thalamus, hypothalamus, hippocampus, amygdala, midbrain, cerebellum, and brainstem were quickly collected and stored at −80°C. Total RNA was extracted using a TaKaRa MiniBest universal RNA Extraction kit (Catalog number: 9767, Takara Bio Inc., Japan) according to the manufacturer’s protocol. The RNA (0.5 µg) was reverse-transcribed into cDNA by using a TaqMan^®^ Reverse Transcription Kit (Catalog number: N8080234; Thermo Fisher Scientific, United States), following the manufacturer’s protocol. Conventional PCR was performed in 15 µl of buffer solution containing 1 µl of template cDNA, 7.5 µl GoTaq^®^ G2 Hot Start Green Master Mix and 10 pmol of each primer. The primers were as follow: NUCB2, forward 5′- TCC​AAT​CCA​TCA​GAT​TCT​TCC-3′ and reverse 5′-CCT​GAC​AAG​TTT​GAG​CCC​AC-3’; GAPDH, forward 5′-ACC​ACA​GTC​CAT​GCC​ATC​AC -3′ and reverse 5′-TCC​ACC​ACC​CTG​TTG​CTG​TA -3’. The optimum temperature cycling protocol was as follow: 95°C for 2 min, 40 cycles with 95°C for 30 s, 56°C for 30 s and 72°C for 1 min, then 72°C for 5 min, using a MiniAmp Plus thermal cycler (Thermo Fisher Scientific Company). The PCR products were run on a 2% agarose gel and visualized with GelRed^®^ Nucleic Acid Gel Stain agent on a Syngene G:BOX Chemi XRQ gel imaging system. The specificity of the PCR product was confirmed by direct sequencing.

### Quantitative Real-Time PCR (qRT-PCR)

To investigate the level of expression of NUCB2 mRNA under *ad libitum*-fed and fasting conditions, animals were randomly divided into two groups of three animals each. In the fed group, animals were given free access to food whereas in the food deprivation group, animals were fasted for 24 h before tissue collection. The level of expression of NUCB2 mRNA was assessed using quantitative real-time PCR. Real-time PCR was performed in an ABI QuantStudio 7 (QS7) Flex Real Time PCR System using TB Green^®^ Premix Ex Taq™ (Catalog number: RR420L; Takara, Bio Inc.,). The optimum temperature cycling protocol was as follow: 48°C for 2 min, 95°C for 10 min, and 95°C for 15 s, 60°C 1 min for 45 cycles. The primers were as follow: NUCB2, forward 5′- TCC​AAT​CCA​TCA​GAT​TCT​TCC-3′ and reverse 5′-CCT​GAC​AAG​TTT​GAG​CCC​AC-3’; GAPDH, forward 5′-ACC​ACA​GTC​CAT​GCC​ATC​AC -3′ and reverse 5′-TCC​ACC​ACC​CTG​TTG​CTG​TA -3’. The NUCB2 mRNA expression was normalized to GAPDH. The relative mRNA expression of NUCB2 was calculated by the 2^-∆∆Ct^ method.

### Western Blot Analysis

The stomach, gut and brain tissues of three *S. murinus* were lysed in sodium dodecyl sulphate (SDS) lysis buffer containing protease inhibitor cocktail tablets (Complete Mini, Roche). The concentration of protein in each extract was measured using a Bio-Rad Protein Assay Kit (Bio-Rad Laboratories, Hercules, CA, United States) according to manufacturer’s instructions. Briefly, 25 μg of tissue was added to 10% polyacrylamide gels which were then transferred to nitrocellulose membranes, blocked for 1 h at room temperature with 5% bovine serum albumin in washing buffer, and then incubated overnight at 4°C with the nesfatin-1 primary antibody (Catalog number: H-003-22, 1:1,000, Phoenix Pharmaceuticals Inc., United States). Membranes were washed for 3 times, 5 min each, and then incubated with anti-rabbit IgG horseradish peroxidase conjugate (1:2000, Thermo Fisher Scientific, United States) for 1 h, and washed again (4 × 5 min) before incubating with a chemiluminescence detection reagent for 5 min. A ChemDoc XRS detection system (Bio-Rad, Milan, Italy) was used to visualize the protein. GAPDH was served as an internal control, and it was similarly detected using a horseradish peroxidase conjugated mouse anti-GAPDH as the primary antibody.

### Immunohistochemistry

For the gut tissues, 5 µm cross sections were collected onto the slides using a freezing microtome. For the brain tissues, frozen tissues were sectioned at 40 μm in the coronal plane using a freezing microtome and were collected as free-floating sections. The brain and gut sections were first incubated with 0.01% H_2_O_2_ at room temperature for 1 h followed by blocking with 1.5% normal goat serum containing 0.3% Triton X-100 in PBS (Vectastain Elite ABC kit, Vector Laboratories, Burlingame, United States) for 1 h. Sections were then incubated with rabbit anti-nesfatin-1 antibody (1:10,000, #H-003-22, Phoenix Pharmaceuticals, United States) for 48 h at 4°C. The sections were subsequently washed and incubated with secondary goat-anti-rabbit antibody (1:200; Vector Laboratories) for 1 h, followed by Vectastain avidin–biotin complex reagent for 1 h (1:100; Vectastain Elite ABC kit, Vector Laboratories, Burlingame, United States). A control for nonspecific binding of the secondary antibody was performed by omitting the nesfatin-1 primary antibody. Nesfatin-1 expression was visualized using a commercially available peroxidase substrate (Vector^®^ VIP kit, Vector Laboratories, Burlingame, United States). The number of nesfatin-1-immunoreactive cells was assessed manually using a Zeiss Axioskop two plus microscope (Carl Zeiss Inc. Thornwood, United States) equipped with a Zeiss Axiocam 2 camera.

### Organ Bath Studies

The segments of the gastric antrum, duodenum, jejunum, ileum, and colon from nine animals were mounted longitudinally under ∼0.5 g resting tension in a 10 ml organ bath filled with Krebs solution (composition in mM: NaCl 118, KCl 4.7, KH2PO4 1.2, MgSO4•7H2O 1.2, CaCl2•2H2O 2.5, NaHCO3 25 and glucose 10) and gassed with 95% O_2_ and 5% CO_2_. The isometric contractions of tissues were recorded using Grass transducers via a MacLab^®^ system (ADInstruments Pty Ltd., New South Wales, Australia) connected to a Power Macintosh G3 computer (Apple Computer, Inc., California, United States). The amplitude and frequency of the contractions was displayed and analysed using an Analytical software (Chart, version 3.5 s/s MacLab^®^, NSW, Australia). The contractile responses were determined by the change of tension (g) before and after the stimuli. The frequency of contractions was measured over a period of 1 min before and after drug exposure. After an equilibration period of 30 min, KCl (120 mM) was added to provide a reference contractile response followed by washout.

#### Effects of Nesfatin-1 on the Contractile Response of the Stomach and Gut Tissues

After 60 min of equilibration, nesfatin-1 (0.3–1,000 pM) was added to the organ bath cumulatively using a 2–3 min dosing schedule. At the end of the experiment, KCl (120 mM) was added again to check the viability and contractility of the tissues.

#### Effects of Nesfatin-1 on the Response of Carbachol-pre-contracted Stomach and Gut Tissues

The stomach and gut tissues were pre-contracted using carbachol (10 μM). After reaching a stable maximum contraction, nesfatin-1 (0.3–1,000 pM) was added cumulatively using a 2–3 min dosing schedule, followed by atropine (10 μM). At the end of each experiment, carbachol was washed out followed by adding KCl (120 mM) to check the viability of the tissues.

#### Effects of Nesfatin-1 on the Response of Electrical Field Stimulated–Stomach and Gut Tissues

After equilibration, the stomach and gut tissues were contracted by electrical field stimulation (EFS) to produce a sub-maximal contraction. The EFS parameters were: train duration, 10 s; voltage, 90 V; pulse width, 1 ms; frequency, 2–32 Hz; interval, 1 min. After reaching a stable contraction, nesfatin-1 (0.3–1,000 pM) was added cumulatively using a 2–3 min dosing schedule. At the end of each experiment, EFS was stopped and KCl (120 mM) was added to check the viability of the gut tissues.

### Stereotaxic Surgery

A total of 30 animals were fasted overnight and then anaesthetized with sodium pentobarbital (40 mg/kg, i. p.) before being placed into a stereotaxic frame equipped with custom-made ear-bars and mouthpieces (David Kopf Instruments, Tujunga, United States). An incision was made in the skin from just behind the nose to the back of the head, and the temporalis muscles on either side of the sagittal crest were displaced. The skull surface in the immediate vicinity of the crest was then cleared of connective tissue. A hole was drilled in the skull according to the following coordinates for the lateral ventricle: 8.2 mm anterior to lambda and 0.9 mm lateral to the midline. A 30-gauge cannula was then inserted into the hole 1.2 mm below the surface of the dura. Animals were then individually housed and allowed a postsurgical recovery period of 7 days before the commencement of the experiment.

### Administration of Drugs

One day prior to experimentation, animals were transferred to the observation room with controlled lighting (15 ± 2 Lux) and habituated individually to clear Perspex observation chambers (21 × 14 × 13 cm^3^). The animals were food deprived 12 h prior to the administration of drugs; water was given *ad libitum* unless otherwise stated. On the day of experiment, animals were centrally administered with nesfatin-1 (1, 5, 25 and 50 pmol, i.c.v.) or saline (5 μl, i.c.v.). The dose range of nesfatin-1 was chosen based on the results of previous studies (Oh et al., 2006; Maejima et al., 2009). Emesis behaviours and locomotor activities were measured for 6 h whereas food and water consumption was measured hourly for 6 h and at 24 h post-administration. Total food and water consumption at each period were measured by calculating the difference in the weight of the pre-weighted food and water before and after each period. Episodes of emesis were characterized by rhythmic abdominal contractions that were associated with either oral expulsion of solid or liquid materials from the gastrointestinal tract (i.e., vomiting) or without the passage of materials (i.e., retching movements). Two consecutive episodes of retching and/or vomiting were considered separate when an animal changed its location in the observation chamber or when the interval between retches and/or vomits exceeded 2 s (Lau et al., 2005). An EthoVision Color Pro system (Version 2.3; Noldus Information Technology, Costerweg, Netherlands) was used to assess the changes in locomotor activity captured by a closed-circuit camera (Panasonic, WV-PC240, China).

### Statistical Analysis

All data are expressed as mean ± s.e.m. Expression of NUCB2 mRNA in fed and fasted states were assessed using unpaired *t*-test. Contractile responses, emetic episodes, locomotor activity data, and food and water intake were analysed using one-way analysis of variance (ANOVA) or two-way ANOVA (for cumulative data) followed by the Bonferroni multiple comparison tests, as appropriate. Latency data to first emetic episode were assessed by a Kruskal–Wallis test followed by Dunn’s multiple comparison tests, as appropriate (GraphPad Prism version 7.0, Inc. Version, California, United States). When an animal failed to exhibit retching and/or vomiting, a latency value equal to the test period observation time (i.e. 6 h) was used to perform the statistical analysis. Differences were considered statistically significant when *p* < 0.05.

## Results

### *In Silico* Analysis of Nesfatin-1 Peptide

**FIGURE 1:**
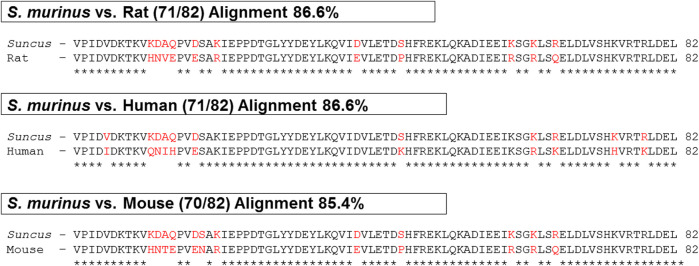
Comparison of nesfatin-1 amino acid sequence in human, rat, mouse and *Suncus murinus*. ∗ (asterisk) denotes positions which have a single, fully conserved residue (space) denotes conservation between groups of similar properties.

The *S. murinus* nesfatin-1 amino acid showed high sequence identity with humans and rodents and shared an 86.6, 86.6 and 85.4% homology with humans, rats, and mice, respectively (Figure 1).

### Expression of NUCB2 mRNA and Nesfatin-1 Protein

**FIGURE 2:**
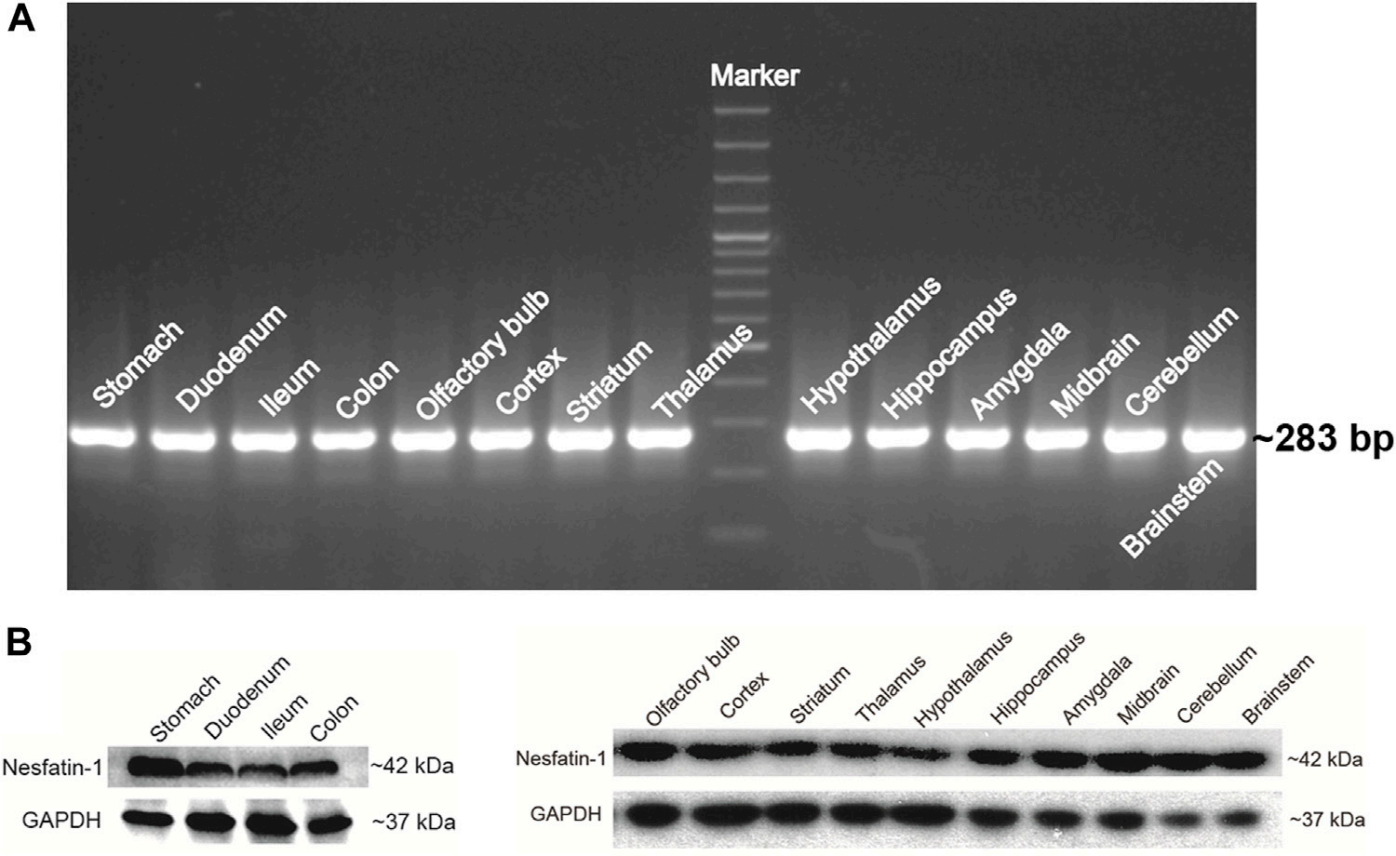
NUCB2/nesfatin-1 expression in various tissues of *Suncus murinus*. **(A)** Conventional PCR analysis of NUCB2 mRNA (∼283 bp) in the stomach, gut and brain. **(B)** Western blot analysis of nesfatin-1 protein (∼42 kDa) in the stomach, gut and brain.

NUCB2 mRNA detected by RT-PCR was expressed in the gastrointestinal tract, including the stomach, duodenum, ileum and colon. NUCB2 mRNA were also detected throughout the entire brain, including the olfactory blub, cortex, striatum, thalamus, hypothalamus, hippocampus, amygdala, midbrain, cerebellum and brainstem (Figure 2A). Western blot analysis confirmed nesfatin-1 protein expression in the stomach, duodenum, ileum and colon and all the brain tissues examined (Figure 2B). Further investigation of the levels of NUCB2 mRNA in *ad libitum*-fed animals and 24 h fasted animals by quantitative RT-PCR showed that the mRNA levels in the hypothalamus, hippocampus and brainstem were significantly decreased (hypothalamus 0.49 ± 0.09 fold, hippocampus 0.44 ± 0.12 fold and brainstem 0.07 ± 0.04 fold) while that in the striatum were increased (2.50 ± 0.34 fold) following food deprivation for 24 h compared to *ad libitum-*fed animals (*p* < 0.05, n = 3), whereas GADPH was not changed. Conversely, the mRNA expression in the gastrointestinal tissues was not affected by food deprivation (Table 1).

**TABLE 1:** The relative NUCB2 mRNA expression levels between 24 h-fasted versus *ad libitum-*fed *S. murinus.*

Tissues	24 h-Fasted versus *ad libitum-*Fed Animals (Fold Change; Mean ± s.e.m.)	*p* Values
Gastrointestinal		
Stomach	1.27 ± 0.53	0.6366
Duodenum	1.30 ± 0.68	0.684
Ileum	2.20 ± 0.89	0.2477
Colon	4.94 ± 1.59	0.0682
Brain		
Olfactory bulb	2.89 ± 1.34	0.2311
Cortex	2.40 ± 1.28	0.3344
Striatum	2.50 ± 0.34	0.011*
Thalamus	6.23 ± 2.92	0.1476
Hypothalamus	0.49 ± 0.09	0.0038**
Hippocampus	0.44 ± 0.12	0.0105*
Amygdala	13.17 ± 12.13	0.3725
Midbrain	0.63 ± 0.39	0.3913
Cerebellum	4.24 ± 3.03	0.3452
Brain stem	0.07 ± 0.04	<0.0001***

The results represent the mean ± s.e.m. of three determinations. Significant differences between animals fasted for 24 h vs *ad libitum*-fed animals are indicated as ∗*p* < 0.05, ∗∗*p* < 0.01, ∗∗∗*p* < 0.001 (Unpaired *t*-test).

### Expression of Nesfatin-1 Immunoreactive Cells

**FIGURE 3:**
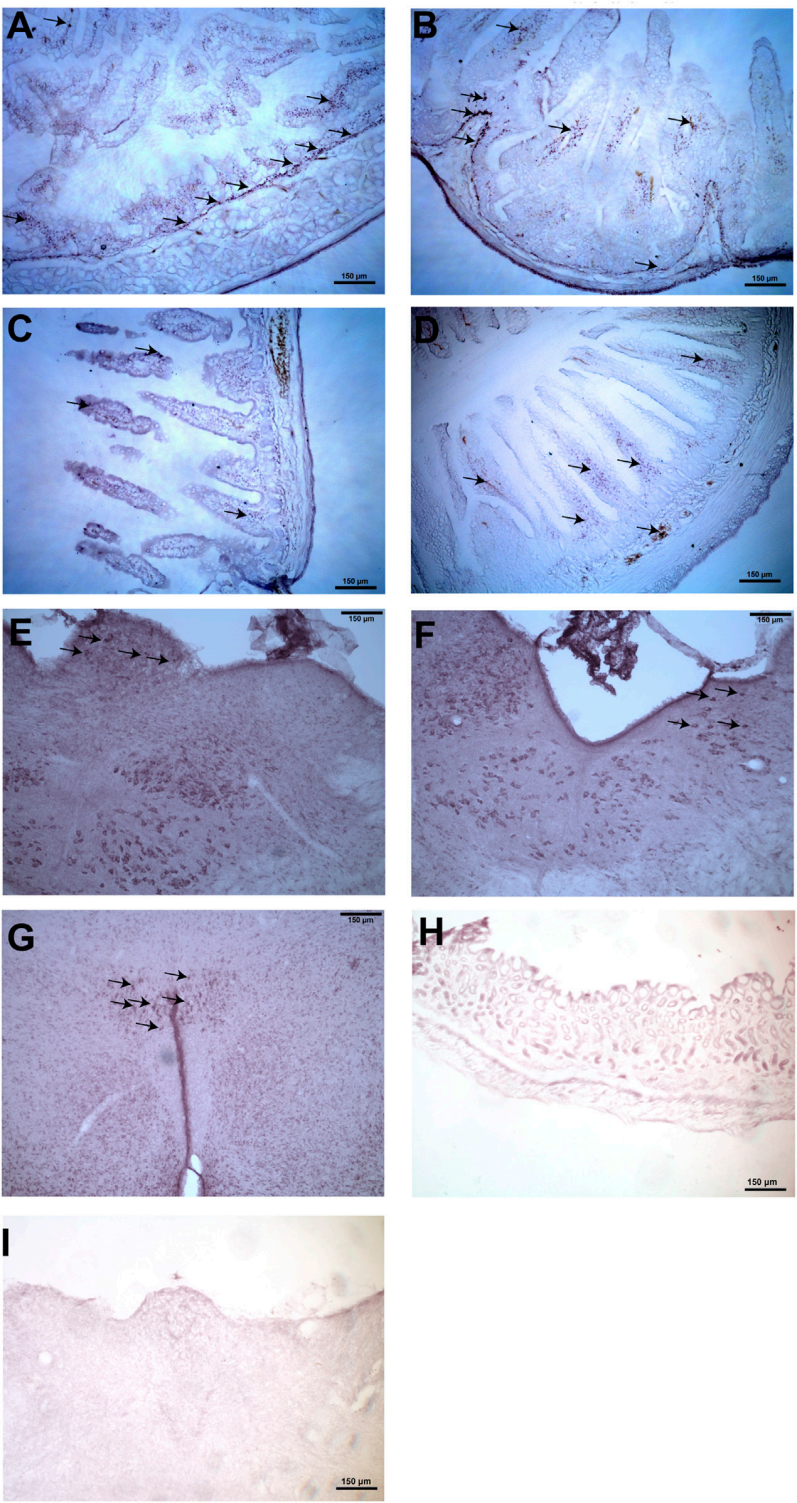
Representative photomicrographs illustrating nesfatin-1 immunoreactive cells (arrows) in the stomach, duodenum, ileum, colon, brainstem and hypothalamus in *Suncus murinus*. **(A)** Nesfatin-1 immunoreactive cells detected in the gastric antrum, **(B)** duodenum, **(C)** ileum, **(D)** colon, **(E)** area postrema, **(F)** nucleus tractus solitarius, and **(G)** hypothalamic paraventricular nucleus. Negative control section in the **(H)** gastric antrum and **(I)** brainstem that omits the primary antibody.

In the stomach, immunohistochemical staining showed that the nesfatin-1 immunoreactive cells was mainly detected in the myenteric plexus of the antrum. Nesfatin-1 immunoreactive cells were also detected in the submucosal plexus and the mucosa region of the antrum, duodenum, ileum and colon (Figure 3). In the brain, nesfatin-1-immunoreactive cells were detected in the brainstem, especially in the DMNV and the NTS. Weaker immunoreactivity were observed at the area postrema (AP). In the forebrain areas, the nesfatin-1 immunoreactive cells were detected in the hypothalamic paraventricular nucleus (PVN) (Figure 3). The staining was absent when the primary antibody was omitted.

### Effects of Nesfatin-1 on the Contractile Response of Isolated Gut Tissues

**FIGURE 4:**
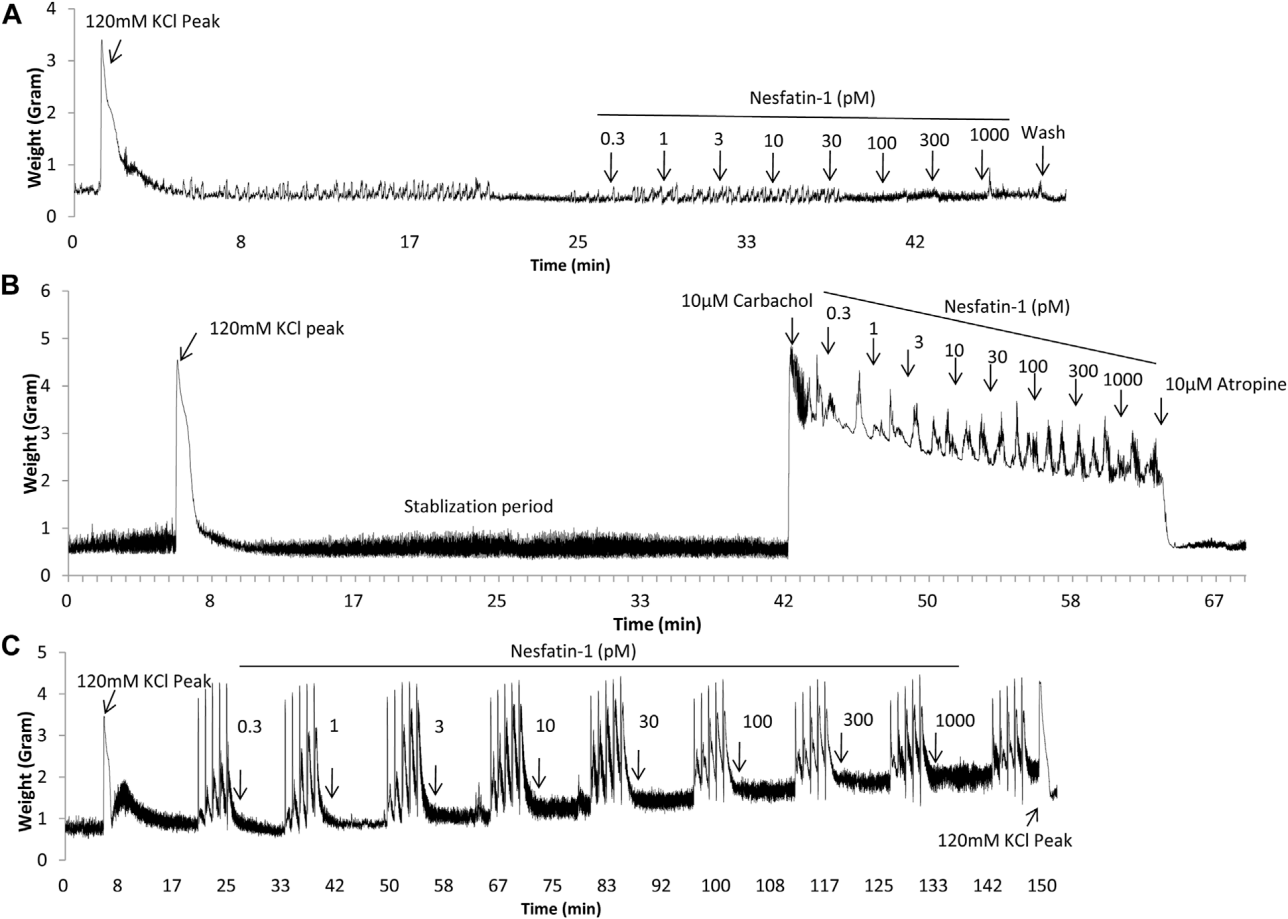
Representative tracings illustrating the contractile responsiveness of *Suncus murinus* duodenal segments to nesfatin-1, carbachol, electrical field stimulation (EFS) and atropine. **(A)** Effect of nesfatin-1 (0.3–1,000 pM), **(B)** effect of nesfatin-1 (0.3–1,000 pM) on the carbachol (10 μM) pre-contracted duodenal segments, and **(C)** effect of nesfatin-1 (0.3–1,000 pM) and atropine (10 μM) on the EFS pre-contracted (2, 4, 8, 16, 32 Hz with 1 min interval) duodenal segments.

Regular spontaneous contractions were observed in the gastric antrum and the segments of duodenum, jejunum, ileum and colon, and the amplitude and frequency are shown in Table 2. Representative traces from duodenal segments are shown in Figure 4. A rapid and reversible contraction in the gastric antrum and gut was induced by 120 mM KCl (data not shown). Nesfatin-1 (0.3–1,000 pmol) failed to affect the spontaneous contractile activity or influence the oscillation frequency of the gastric antrum and gut tissues (n = 3). In another set experiments, carbachol (10 µM) was used to induce a contraction of the gastric antrum and gut to determine if nesfatin-1 has a potential to induce relaxation of the gastric antrum and gut. The results showed that carbachol induced a sustained contraction but nesfatin-1 (0.3–1,000 pmol) failed to affect the carbachol-induced contraction (n = 3). Conversely, atropine (10 µM) significantly reduced the amplitude of carbachol-induced contraction by 82.9, 75.4, 68.5, 70.9 and 75.8% in the isolated segments of antrum (*p* = 0.15), duodenum (*p* < 0.01), jejunum (*p* < 0.05), ileum (*p* < 0.01), and colon (*p* < 0.01), respectively (n = 3). In further experiments, the potential effect of nesfatin-1 on electrical field stimulated contractions of the gastric antrum and gut was examined. EFS-induced contractions in a frequency-dependent manner but nesfatin-1 (0.3–1,000 pmol) was without effect on EFS-induced contractions (n = 3).

**TABLE 2:** Summary of the contraction frequency and amplitude of the gastrointestinal tract tissues *in vitro*.

	Frequency of spontaneous Contractions (Cycles/min) (n = 9)	Amplitude of spontaneous Contractions (g) (n = 9)	Amplitude of KCl Response (g) (n = 9)	Amplitude of carbachol Response (g) (n = 3)	Amplitude of EFS-Induced Response (g) (n = 3)				
					2 Hz	4 Hz	8 Hz	16 Hz	32 Hz
Gastric antrum	12.2 ± 0.7	1.44 ± 0.17	4.33 ± 0.54	3.43 ± 1.05	3.52 ± 0.46	4.76 ± 0.23	5.08 ± 0.34	5.55 ± 0.51	5.14 ± 0.52
Duodenum	32.6 ± 2.2	0.71 ± 0.08	3.19 ± 0.28	3.48 ± 0.16	2.86 ± 0.54	3.13 ± 0.54	3.13 ± 0.59	3.13 ± 0.59	3.02 ± 0.62
Jejunum	28.6 ± 2.0	0.66 ± 0.07	3.93 ± 0.24	3.78 ± 0.40	3.13 ± 0.60	3.28 ± 0.63	3.44 ± 0.63	3.64 ± 0.63	3.71 ± 0.65
Ileum	26.8 ± 1.8	0.64 ± 0.06	3.74 ± 0.30	3.26 ± 0.84	3.05 ± 0.82	4.09 ± 0.55	4.45 ± 0.53	4.58 ± 0.64	4.56 ± 0.61
Colon	28.3 ± 1.5	0.81 ± 0.17	4.94 ± 0.39	5.41 ± 1.14	4.51 ± 1.13	5.41 ± 0.69	5.88 ± 0.83	6.02 ± 1.01	5.95 ± 1.02

Data represents the mean ± s.e.m.

### Effects of Centrally Administered Nesfatin-1 on Emesis

**FIGURE 5:**
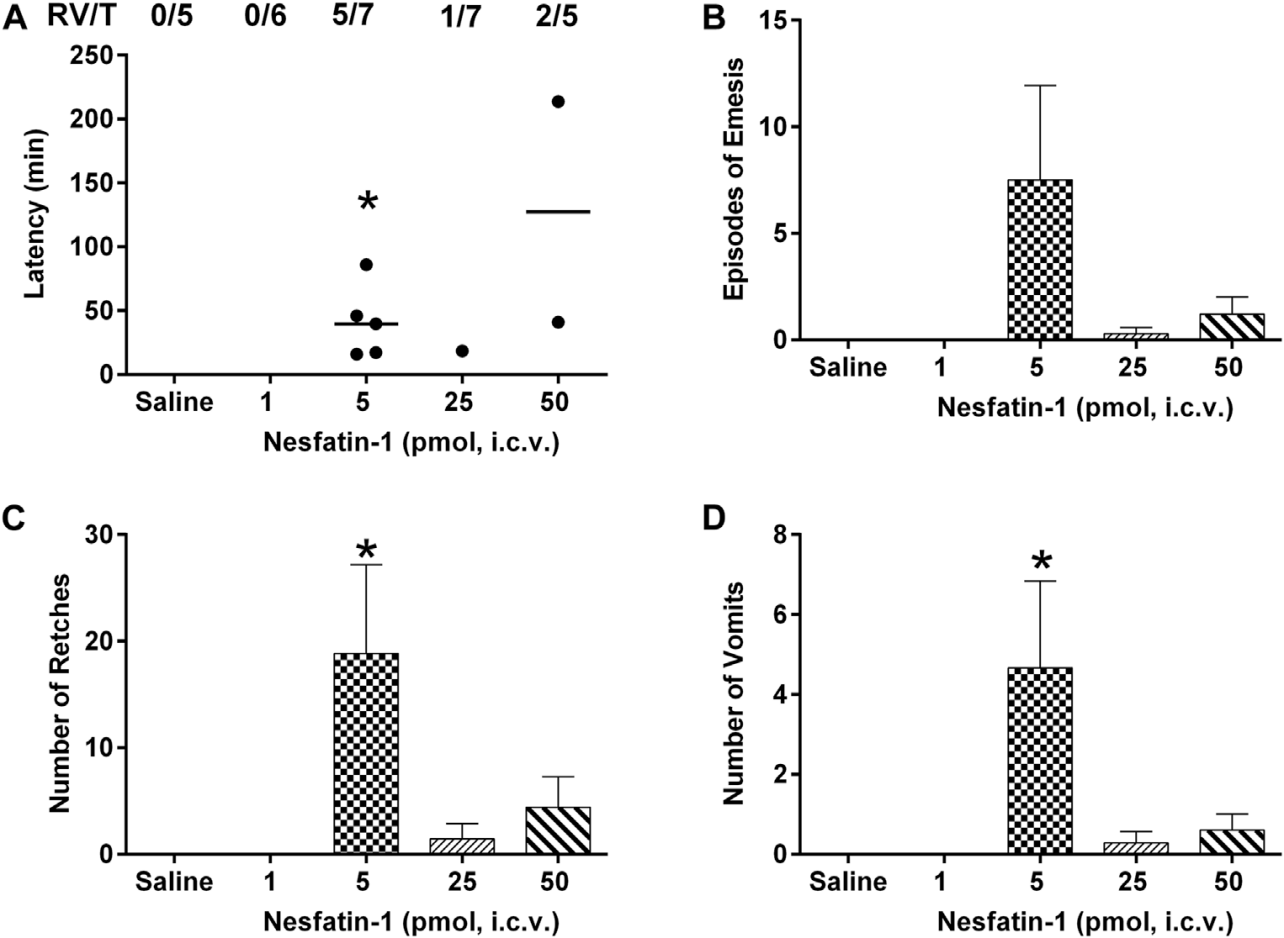
Effects of intracerebral drug administration on emesis in *Suncus murinus*. **(A)** Latency to emesis, **(B)** episodes of emesis, **(C)** number of retches and **(D)** number of vomits after an i.c.v. administration of saline (5 μl, i.c.v.) or nesfatin-1 (1–50 pmol, i.c.v). The results represent the mean ± s.e.m. of 5–7 determinations during a period of 6 h. The number of animals retching and/or vomiting out of the number of animals tested (RV/T) is indicated as a fraction of each treatment group. Latency data are shown as individual responses, with lines indicating the median response time. Significant differences between the treatment groups are indicated as ∗*p* < 0.05 (one-way ANOVA followed by Bonferroni test).

The intracerebral administration of saline was not associated with retching or vomiting. Nesfatin-1 at 1 pmol, i.c.v., failed to induce retching or vomiting (n = 5–7; Figure 5A –5D). However, nesfatin-1 at 5 pmol, i.c.v., induced emesis in five out of seven animals with 18.8 ± 8.3 retches and 4.7 ± 2.1 vomits following a median latency of 39.7 min (25th percentile =; 75th percentile = min; *p* < 0.05; n = 5–7; Figure 5A–5D). Nesfatin-1 at 25 and 50 pmol, i.c.v., induced emesis in one out of seven and two out of five animals, respectively, within 18.5–214 min (Figure 5A–5D; *p* > 0.05).

### Effects of Centrally Administered Nesfatin-1 on Food Intake

**FIGURE 6:**
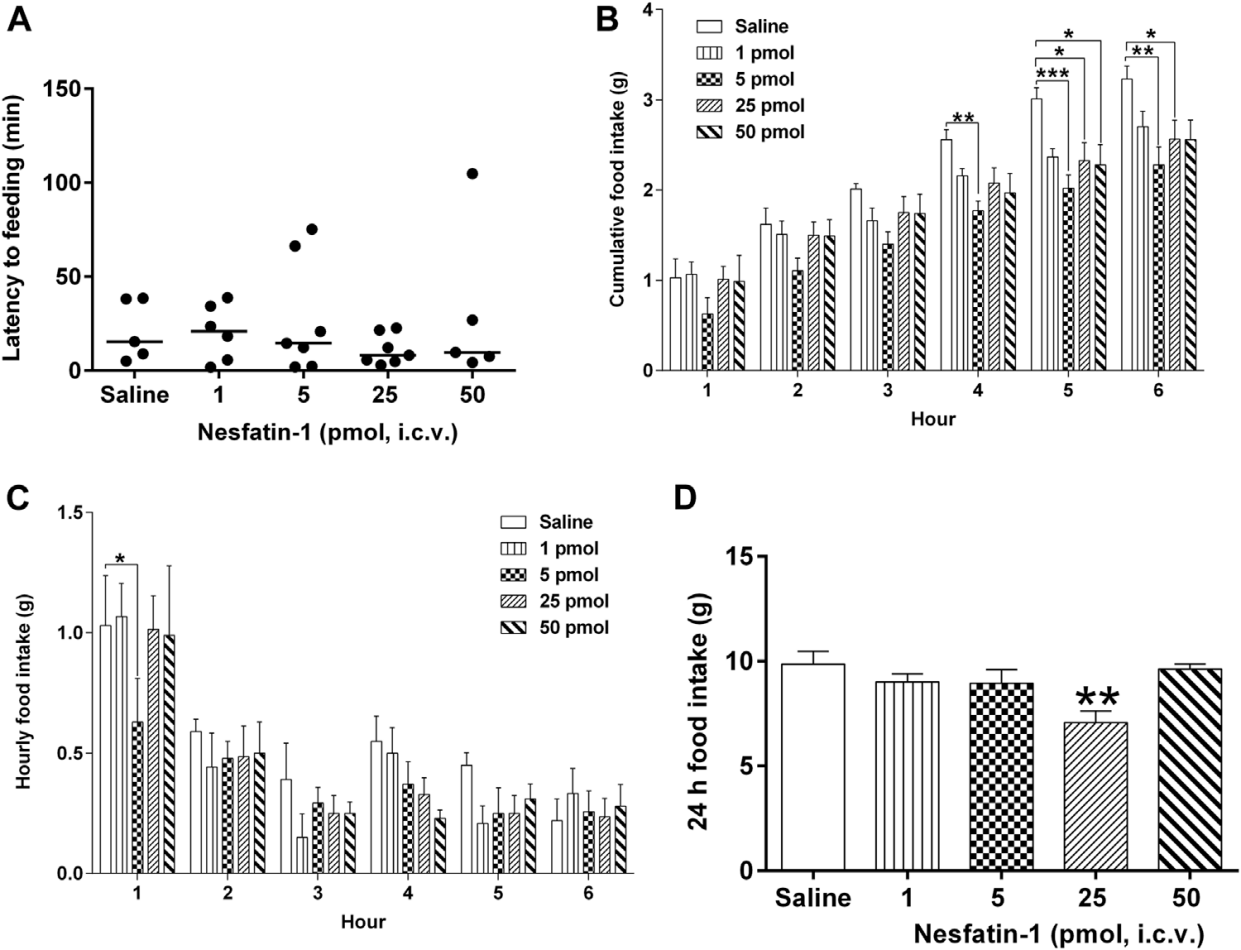
Effects of intracerebral drug administration on food intake in *Suncus murinus*. **(A)** Latency to food intake, **(B)** cumulative food intake from 0 to 6 h, **(C)** hourly food intake from 0–6 h, and **(D)** cumulative food intake at 24 h after an i.c.v. administration of saline (5 μl, i.c.v.) or nesfatin-1 (1–50 pmol, i. c.v). The results represent the mean ± s. e.m. of 5–7 determinations during a period of 24 h. Latency data are shown as individual responses, with lines indicating the median response time. Significant differences between the treatment groups are indicated as ∗*p* < 0.05, ∗∗*p* < 0.01 and ∗∗∗*p* < 0.001 (one-way ANOVA or two-way ANOVA, followed by Bonferroni test).

Central administration of nesfatin-1 (1–50 pmol) had no effect on the latency to food intake (Figure 6A). Nesfatin-1 at 5 pmol, i.c.v. significantly reduced the 4-, five- and 6-h cumulative food intake by 30.9, 32.9, and 29.4%, respectively, compared to saline-treated animals (n = 5–seven; *p* < 0.01; Figure 6B), while at 25 pmol, nesfatin-1 reduced the five- and 6-h cumulative food intake by 22.6 and 20.6%, respectively (*p* < 0.05; Figure 6B). Higher dose of nesfatin-1 (50 pmol, i.c.v.) only caused a significant reduction (24.3%) at the 5-h cumulative food intake (*p* < 0.05; Figure 6B). Regarding hourly food intake, nesfatin-1 at 5 pmol, i.c.v. significantly reduced food intake by 39.0% at the first hour (*p* < 0.05; Figure 6C). However, the hourly reduction in food intake was no longer different between nesfatin-1-treated and saline-treated animals at second to sixth hour (*p* > 0.05; Figure 6C). At 24 h post-administration, nesfatin-1 at 25 pm,ol, i.c.v. produced a significant 28.3% reduction in food intake (*p* < 0.01; Figure 6D).

### Effects of Centrally Administered Nesfatin-1 on Water Intake

**FIGURE 7:**
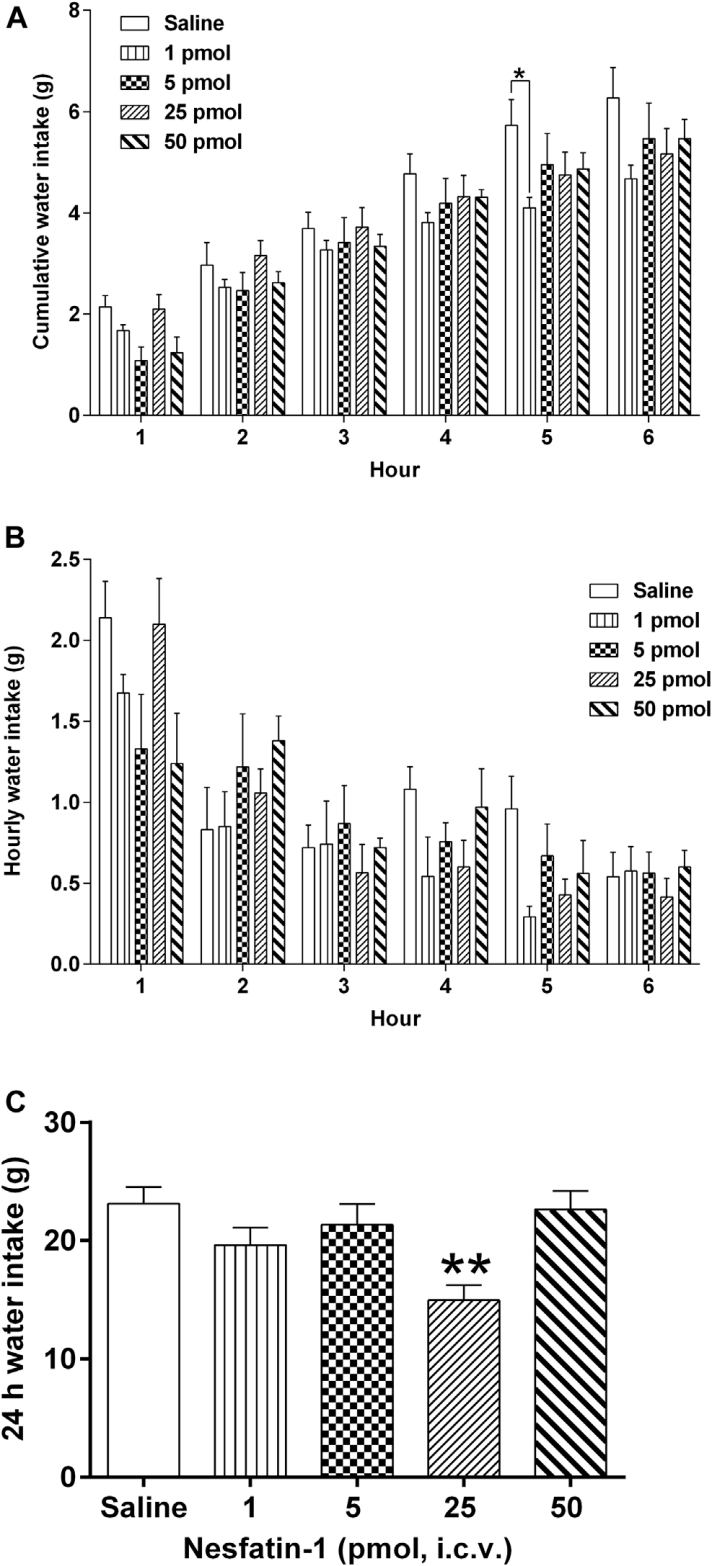
Effects of intracerebral drug administration on water intake in *Suncus murinus*. **(A)** Cumulative water intake from 0 to 6 h, **(B)** hourly water intake from 0–6 h, and **(C)** cumulative water intake at 24 h after an i.c.v. administration of saline (5 μl, i.c.v.) or nesfatin-1 (1–50 pmol, i.c.v). The results represent the mean ± s. e.m. of 5–7 determinations during a period of 24 h. Significant differences between the treatment groups are indicated as ∗*p* < 0.05 and ∗∗*p* < 0.01 (one-way ANOVA or two-way ANOVA, followed by Bonferroni test).

Central administration of nesfatin-1 (1–50 pmol) had no effect on hourly water intake (Figure 7B). Nesfatin-1 at 1 pmol, i.c.v., significantly reduced the 5-h cumulative water intake by 28.4% (*p* < 0.05; Figure 7A), however, higher doses (5–50 pmol, i.c.v.) had no effect. In addition, nesfatin-1 at 25 pmol, i.c.v., reduced the 24-h cumulative water intake by 35.4% (*p* < 0.01; Figure 7C).

### Effects of Centrally Administered Nesfatin-1 on Locomotor Activity

**FIGURE 8:**
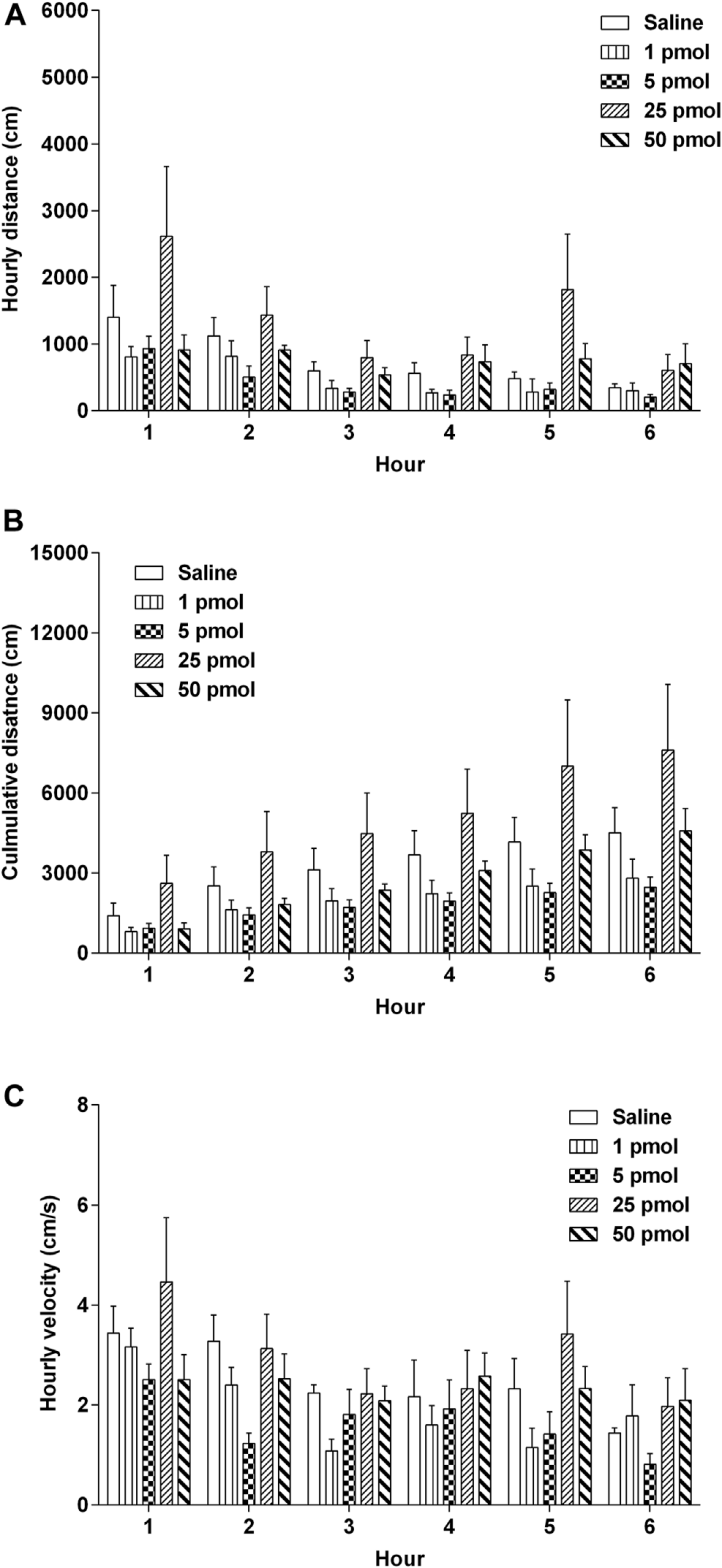
Effects of intracerebral drug administration on locomotor activity in *Suncus murinus*. **(A)** Hourly distance travelled, **(B)** cumulative distance travelled, and **(C)** hourly velocity from 0–6 h an i.c.v. administration of saline (5 μl, i.c.v.) or nesfatin-1 (1–50 pmol, i.c.v.). The results represent the mean ± s. e.m. of 5–7 determinations during a period of 6 h. No significant differences between the treatment groups are observed (one-way ANOVA or two-way ANOVA, followed by Bonferroni test).

The locomotor activity (distance travelled and velocity) of the animals was evaluated by the Nodus tracking software. Nesfatin-1 (1–50 pmol, i.c.v.) had no significant effects on the hourly distance travelled, cumulative distance travelled or hourly velocity of movement or activity (Figure 8A–8C).

## Discussion

The present study identified for the first time the amino acid and cDNA sequence, tissue expression and biological activity of NUCB2/nesfatin-1 in *S. murinus*. We found that the amino acid sequence of nesfatin-1 is highly conserved among humans, rats, mice and *S. murinus*. NUCB2 mRNA and nesfatin-1 protein are abundantly expressed in the brain, stomach and gut. The expression of NUCB2 mRNA in the hypothalamus, hippocampus and brainstem were significantly decreased whereas that in the striatum were increased following food deprivation for 24 h. Nesfatin-1 has no effect on gastrointestinal contractility. Nevertheless, intracerebroventricular administration of nesfatin-1 induces emesis and inhibits food and water intake for up to 24 h, without any effect on locomotor activity.

Previous studies demonstrated that NUCB2, which encodes a 24-amino acid signal peptide and a protein structure containing 396 amino acids, is highly conserved across mammalian and non-mammalian vertebrates (Oh et al., 2006; Mohan and Unniappan, 2013). Using *in silico* cloning, we confirmed that nesfatin-1 amino acid sequence in *S. murinus* shared high homology with the human, rat and mouse genome. The 30-amino acid mid-segment of nesfatin-1, which is considered to be the bioactive core of the protein, is highly conserved across these species (Mohan and Unniappan, 2013). The high sequence identity suggests that this peptide has significant phylogenetic and physiological implications.

In the brain, nesfatin-1 protein is produced in the hypothalamus, including the PVN and the arcuate nucleus (Oh et al., 2006; Stengel et al., 2011; Goebel-Stengel and Wang, 2013) whereas the stomach is the primary peripheral source (Prinz et al., 2016). Nesfatin-1 immunoreactive neurons co-localize with several neurotransmitters (Foo et al., 2008; Inhoff et al., 2010; Goebel-Stengel and Wang, 2013), suggesting that nesfatin-1 may interact with various transmitters in mechanism controlling feeding, endocrine and autonomic functions. Our results showed that NUCB2 mRNA and nesfatin-1 protein were detected in the brain and the gastrointestinal tissues analyzed. In the CNS, nesfatin-1 immunoreactivity was centered at the brainstem and paraventricular hypothalamus and these data were in line with that in rodents (Foo et al., 2008; Stengel et al., 2009; Zhang et al., 2010). We also provided new finding by demonstrating nesfatin-1 immunoreactivity at the AP and in the duodenum, ileum and colon.

It has been demonstrated that fasting decreased the hypothalamic NUCB2 mRNA (Oh et al., 2006; Kohno et al., 2008), whereas refeeding increased the NUCB2 mRNA in the supraoptic nucleus (SON), which was accompanied by an activation of NUCB2/nesfatin-1-positive neurons in the PVN and SON (Kohno et al., 2008). Our results indicated that the mRNA levels in the hypothalamus, hippocampus and brainstem were significantly decreased whereas that in the striatum were increased following food deprivation for 24 h compared to control group. The down-regulation of NUCB2 mRNA in the hypothalamus and brainstem following 24 h food deprivation are compatible with the proposed role of nesfatin-1 as an anorectic peptide and that NUCB2 gene expression may be regulated by nutritional status (Stengel et al., 2011). Hippocampus is a complex brain region that plays a major role in learning and memory (Squire, 1992) and is associated with the modulation of anxiety states (Engin et al., 2016). We observed an up-regulation of NUCB2 mRNA in the striatum. It has been speculated that striatum may be engaged in inducing the effect of nesfatin-1 on the reward value of food (Dore et al., 2020). Our data revealed that nesfatin-1 might be involved in emotional change during fasting. The role of nesfatin-1 as a link between hippocampus, striatum, hypothalamus and brainstem requires further investigation.

Nesfatin-1 was shown to modulate gastric emptying (Stengel et al., 2009; Goebel-Stengel et al., 2011; Tian et al., 2014) and antroduodenal motility (Atsuchi et al., 2010), probably act via central pathway (Guo et al., 2015). In the present studies, nesfatin-1 failed to either contract or relax isolated gastric antrum and intestinal tissues even though we detected nesfatin-1 immunoreactive cells in these tissues.

Our finding regarding the emetic potential of nesfatin-1 is of particular interest. Nesfatin-1 at 5 pmol, i.c.v., induced emesis in 83% animals whereas nesfatin-1 at higher doses, i.e. 25 and 50 pmol appeared to have a lower efficacy in inducing emesis. A recent study reported that central administration of nesfatin-1 decreases the reward value of sucrose but did not induce taste aversion and/or malaise, nor reduces appetite (Dore et al., 2020). In line with this finding, intraperitoneal injection of nesfatin-1 reduces dark-phase food intake without causing conditioned taste aversion in mice (Shimizu et al., 2009). However, intraperitoneal injection of cholecystokinin (CCK), which induces nauseagenic response, causes release of vasopressin with an activation of nesfatinergic neurons in the PVN and NTS (Noetzel et al., 2009; Stengel et al., 2009). Furthermore, gastric electrical stimulation, an alternative therapy to treat patients with intractable vomiting, reduces significantly the levels of NUCB2/nesfatin-1 in fasted patients (Meleine et al., 2017), suggesting that NUCB2/nesfatin-1 may be involved in mechanism of vomiting. To the best of our knowledge, nesfatin-1 is the most potent compound to induce emesis in *S. murinus*, followed by the GLP-1 receptor agonist exendin-4. In our previous studies, the minimum dose of exendin-4 required to induce emesis following intracerebroventricular administration was 100 pmol (Chan et al., 2013), which is 20 times higher than the lowest emetic dose of nesfatin-1.

Central administration of nesfatin-1 reduces dark-phase food intake in rats (Oh et al., 2006; Stengel et al., 2011) via leptin-independent melanocortin pathway (Maejima et al., 2009), whereas blockade of endogenous nesfatin-1 with an antisense oligonucleotide stimulates food intake (Oh et al., 2006). In the present study, we showed that administration of nesfatin-1 (1–50 pmol) into the lateral ventricle reduced food intake in *S. murinus* fasted for 12 h. Equivalent doses of nesfatin-1 have been shown to reduce dark-phase food intake in rodents (Oh et al., 2006; Stengel et al., 2009). In *S. murinus*, nesfatin-1 at 5 pmol, i.c.v., appeared to be the most potent in inhibiting cumulative food intake in the first 6 hours. However, a higher dose, i.e., 25 pmol, i.c.v. was required to produce significant effect at 24 h post-administration.

In addition to inhibition of food intake, intracerebroventricular administration of nesfatin-1 was shown to reduce water intake and the effect appears to be independent of its anorexigenic action (Yosten and Samson, 2009; Yosten et al., 2012; Yoshimura et al., 2014). We didn’t observe a significant effect of nesfatin-1 on water intake in the first 6 hour, however, nesfatin-1 at 25 pmol, i.c.v. inhibited significantly 24 h water intake and the effect on inhibiting water intake was more pronounce than the anorexigenic effect, i.e., 35.4% reduction in water intake vs 28.3% reduction in food intake. Our data provide evidence that the effects of nesfatin-1 on water intake may be partly independent of its anorexigenic action.

There are some limitations of the present study. Thus far, the identity of the nesfatin-1 receptor remains unknown (Schalla and Stengel, 2018), making it difficult to ascertain the exact site of the action of nesfatin-1. The present studies did not investigate the mechanism of action of nesfatin-1 in inhibiting food and water intake and inducing emesis and it is not clear if peripheral administration of nesfatin-1 inhibit food and water intake and induce emesis in *S. murinus*. Nesfatin-1 immunoreactive neurons co-localize with a number of neurotransmitters including vasopressin in rodents (Goebel-Stengel and Wang, 2013). Further, it is known that pre-treatment with a nesfatin-1 antisense oligonucleotide reduces levels of nesfatin-1, but not vasopressin in the hypothalamic paraventricular nucleus, resulting in an increased drinking response following angiotensin II (Yosten et al., 2012). However, a high-salt diet-induced elevation of plasma vasopressin and vasopressin mRNA in the PVN are blunted by PVN-specific NUCB2 knockdown (Nakata et al., 2016). The inhibitory effect of nesfatin-1 on water intake may involve the release of vasopressin, which has been implicated in the regulation of fluid homeostasis and as a biomarker for nausea and emesis (Cubeddu et al., 1990). Whilst nesfatin-1 failed to modulate the contractility of the gastrointestinal segments in the *in vitro* studies, however, we cannot exclude the involvement of reduced gastric emptying in inhibiting food and water intake. In rats, gastric distension induced c-Fos expression in NTS NUCB2/nesfatinergic neurons (Bonnet et al., 2013), whereas administration of nesfatin-1 into the ventromedial hypothalamus decreases the firing rate of gastric distension excitatory neurons and increases the firing rate of gastric distension inhibitory neurons via an interaction with the CRF signaling pathway (Feng et al., 2017). It appears that a central nesfatin-1 signaling pathway may play a role in the regulation of gastric motility but this could not be replicated using gastrointestinal segments and *in vitro* experimentation. In the present study, interestingly, no clear dose-response relationship was observed. We tested doses at 5 times difference and it is not known if a dose between 5 and 25 pmol lies on the descending limb of the dose-response effect. The lower efficacy of the higher dose of nesfatin-1 to inhibit food and water intake and induce emesis could be due to receptor desensitization or by activation of other unspecified targets that are inhibitory, but this requires further investigation. The former explanation may be more likely, as in streptozotocin-induced type 2 diabetic mice, a low dose of nesfatin-1 activates the AMPK-ACC pathway more effectively than a higher dose (Dong et al., 2013). Other studies have suggested that the nesfatin-1 singling pathway is via a G_i_ protein-coupled receptor (Rupp et al., 2021) which can be desensitized following activation by regulatory peptides, such as ghrelin (Camina et al., 2004) and substance P (Holland et al., 1993).

## Conclusion

In conclusion, NUCB2/nesfatin-1 is highly conserved in *S. murinus*, human, rat and mice. NUCB2/nesfatin-1 might be a potent regulator involved in emesis control, metabolic homeostasis and appetite in *S. murinus*. Further studies are required to elucidate the mechanism of actions of this peptide as a mediator linking the brainstem NUCB2/nesfatin-1 to forebrain system.

## Data Availability

The original contributions presented in the study are included in the article/Supplementary Material, further inquiries can be directed to the corresponding author.
